# RETRACTION: Thyroxine Affects Lipopolysaccharide‐Induced Macrophage Differentiation and Myocardial Cell Apoptosis via the NF‐κB P65 Pathway Both In Vitro and In Vivo

**DOI:** 10.1155/mi/9865458

**Published:** 2026-05-20

**Authors:** Mediators of Inflammation

RETRACTION: S. Zhu, Y. Wang, H. Liu, et al., “Thyroxine Affects Lipopolysaccharide‐Induced Macrophage Differentiation and Myocardial Cell Apoptosis via the NF‐κB P65 Pathway Both In Vitro and In Vivo,” *Mediators of Inflammation* 2019, no. 1 (2019): 2098972, https://doi.org/10.1155/2019/2098972.

The above article, published online on 14 May 2019 in Wiley Online Library (wileyonlinelibrary.com), has been retracted by agreement between the journal Editor‐in‐Chief, Anshu Agrawal; and John Wiley & Sons Ltd. The retraction has been agreed after an investigation into image concerns raised by *Aneurus inconstans* via PubPeer [1].

Specifically, the blot representing Bax in Figure 4a shares unexpected similarities with the blot representing p‐AMPK in Figure 6c of another article authored by the same group [2].

The authors initially requested a correction, but they could not provide their raw data due to technical issues. The authors then later became unresponsive to the journal’s requests for their original, unedited images.

As such, the Chief Editor and Publisher believe that the results cannot be considered as reliable. Therefore, this article is retracted.

The authors were notified of this decision, but remained unresponsive.
